# Improved Visibility of Early Gastric Cancer after Successful *Helicobacter pylori* Eradication with Image-Enhanced Endoscopy: A Multi-Institutional Study Using Video Clips

**DOI:** 10.3390/jcm10163649

**Published:** 2021-08-18

**Authors:** Shinya Matsumura, Osamu Dohi, Nobuhisa Yamada, Akihito Harusato, Takeshi Yasuda, Takuma Yoshida, Tsugitaka Ishida, Yuka Azuma, Hiroaki Kitae, Toshifumi Doi, Ryohei Hirose, Ken Inoue, Naohisa Yoshida, Kazuhiro Kamada, Kazuhiko Uchiyama, Tomohisa Takagi, Takeshi Ishikawa, Hideyuki Konishi, Yukiko Morinaga, Mitsuo Kishimoto, Nobuaki Yagi, Yuji Naito, Yoshito Itoh

**Affiliations:** 1 Department of Molecular Gastroenterology and Hepatology, Graduate School of Medical Science, Kyoto Prefectural University of Medicine, Kyoto 602-8566, Japan; matsumu@koto.kpu-m.ac.jp (S.M.); t-yasuda@koto.kpu-m.ac.jp (T.Y.); takuma-y@koto.kpu-m.ac.jp (T.Y.); t-ishida@koto.kpu-m.ac.jp (T.I.); ayuka@koto.kpu-m.ac.jp (Y.A.); hkitae@koto.kpu-m.ac.jp (H.K.); t-doi@koto.kpu-m.ac.jp (T.D.); ryo-hiro@koto.kpu-m.ac.jp (R.H.); keninoue71@koto.kpu-m.ac.jp (K.I.); naohisa@koto.kpu-m.ac.jp (N.Y.); k-kamada@koto.kpu-m.ac.jp (K.K.); k-uchi@koto.kpu-m.ac.jp (K.U.); takatomo@koto.kpu-m.ac.jp (T.T.); iskw-t@koto.kpu-m.ac.jp (T.I.); hkonishi@koto.kpu-m.ac.jp (H.K.); ynaito@koto.kpu-m.ac.jp (Y.N.); yitoh@koto.kpu-m.ac.jp (Y.I.); 2 Department of Gastroenterology, Matsushita Memorial Hospital, Osaka 570-8540, Japan; nobukpum@gmail.com; 3 Department of Gastroenterology, North Medical Center, Kyoto Prefectural University of Medicine, Kyoto 629-2261, Japan; harup@koto.kpu-m.ac.jp; 4 Department of Surgical Pathology, Graduate School of Medical Science, Kyoto Prefectural University of Medicine, Kyoto 602-8566, Japan; myukiko@koto.kpu-m.ac.jp; 5 Department of Pathology, Kyoto City Hospital, Kyoto 604-8845, Japan; kish-pat@koto.kpu-m.ac.jp; 6 Department of Gastroenterology, Asahi University Hospital, Gifu 501-0223, Japan; nyagi@koto.kpu-m.ac.jp

**Keywords:** gastric cancer, *Helicobacter pylori*, linked color imaging, blue laser imaging, Kyoto classification of gastritis

## Abstract

The visibility and diagnostic accuracy of early gastric cancer (EGC) after *Helicobacter pylori* (HP) eradication have been reported to improve using image-enhanced endoscopy (IEE) compared with white light imaging (WLI). The present study clarified the appropriate IEE for the detection and diagnosis of EGC in clinical settings. This prospective and cross-sectional study evaluated the visibility of EGC and endoscopic findings of gastric mucosa after successful HP eradication (*n* = 31) using videos with WLI and IEE. Three endoscopists evaluated high-definition videos in a randomized order. The mean visibility scores (MVSs) on linked color imaging (LCI) for atrophic border, intestinal metaplasia, map-like redness, and EGC were the highest among each modality (3.87 ± 0.34, 3.82 ± 0.49, 3.87 ± 0.50, and 3.35 ± 0.92, respectively). The MVSs with blue laser imaging (BLI) were highest for magnifying view of the demarcation line (DL), microsurface pattern (MSP), and microvascular pattern (MVP) for EGC (3.77 ± 0.49, 3.94 ± 0.25, and 3.92 ± 0.34, respectively). LCI had the highest visibility among findings of gastric mucosa and EGC after HP eradication, and BLI had the highest visibility of MVP, MSP, and DL in magnifying observation. These results suggest that LCI observation in the entire stomach and further magnifying BLI are the best methods for detecting and diagnosing EGCs after HP eradication, respectively.

## 1. Introduction

Eradication therapy against *Helicobacter pylori* (HP) infection, which is defined as an obvious carcinogen [1], has been recommended for the prevention of gastric cancer (GC) [2]. However, GC often occurs even after successful HP eradication [3,4,5], and it is often difficult to detect and diagnose early GC (EGC) after HP eradication correctly using white light imaging (WLI) [6].

The visibility and diagnostic accuracy of EGC have been reported to be improved using image-enhanced endoscopy (IEE) modalities with laser light sources, including blue laser imaging (BLI) [7,8,9], and linked color imaging (LCI) [10,11,12,13], compared with WLI. Furthermore, BLI and LCI show enhanced endoscopic findings of HP-related gastritis, including diffuse redness, intestinal metaplasia, atrophic border, and map-like redness, compared with WLI [14,15,16,17,18,19]. However, there is a clinical question regarding which IEE should be applied during observation of the stomach, as which is more suitable for evaluating the visibility of EGC and endoscopic findings after HP eradication during screening endoscopy remains unclear.

The present study intended to clarify the appropriate IEE between BLI and LCI for visibility of GC and background mucosa after HP eradication in a clinical setting.

## 2. Materials and Methods

### 2.1. Participating Institutions

This prospective and cross-sectional study was conducted at four medical institutions including the hospital of Kyoto Prefectural University of Medicine, North Medical Center, Kyoto Prefectural University of Medicine, Asahi University Hospital, and Matsushita Memorial Hospital. This study was approved by the Ethical Review Committee of Kyoto Prefectural University of Medicine (ERB-C-875) and the other three institutions, and it was carried out in accordance with the World Medical Association’s Helsinki Declaration. The protocol was registered with the University Hospital Medicine Information Network (registration number UMIN000026491).

### 2.2. Patients

The patients scheduled to undergo endoscopic submucosal dissection (ESD) for EGC after successful HP eradication from September 2017 to April 2019 in the 4 institutions were prospectively enrolled in this study. Patients with EGC who were scheduled to undergo ESD were assessed for eligibility.

The inclusion criterion was the presence of EGC after successful HP eradication. The exclusion criteria were as follows: multiple GCs, lesions less than 10 mm in size, recurrent GC, post-operated stomach, <1 year after HP eradication therapy, EGCs with white coat mucus or ulceration, and endoscopic clip or tattooing around the lesion that could affect lesion recognition. All patients provided their written informed consent for enrollment in this study.

### 2.3. Outcomes

The primary endpoint of the study was the visibility of EGC after successful HP eradication. The secondary endpoint was the visibility of endoscopic findings of the Kyoto classification of gastritis after successful HP eradication.

### 2.4. Endoscopic System, Devices, and Settings for the Movies

**Figure 1:**
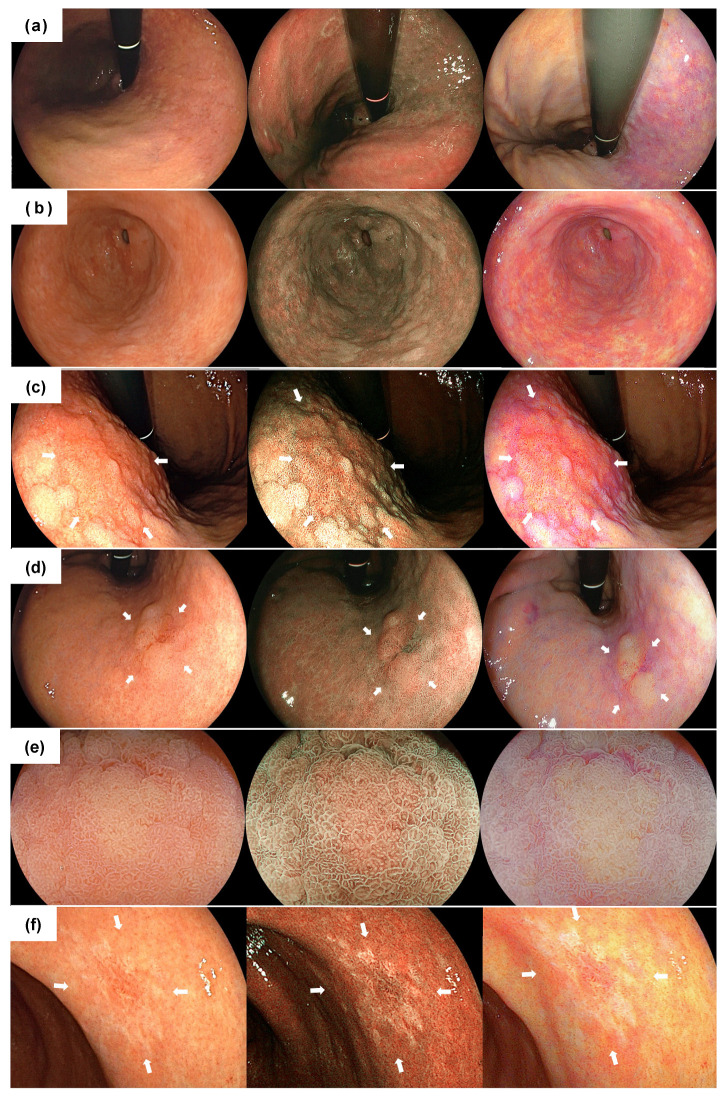
Typical endoscopic images of each finding using white light imaging (**left**), blue laser imaging (**center**) and linked color imaging (**right**). (**a**) distant view of the atrophic border; (**b**) distant view of the intestinal metaplasia; (**c**) distant view of the differentiated adenocarcinoma (27 mm size, type 0-II c) surrounded by map-like redness. The lesion is indicated by white arrows; (**d**) non-magnifying image of the differentiated adenocarcinoma (11 mm size, type 0-IIa,). The lesion is indicated by white arrows; (**e**) magnifying image of the lesion; (**f**) non-magnifying image of the undifferentiated adenocarcinoma (12 mm size, type 0–II c). The lesion is indicated by white arrows.

All examinations were carried out by 4 skilled endoscopists accredited by the Japan Gastroenterological Endoscopy Society, with EG-L590ZW and EG-L600ZW endoscopes corresponding to the LASEREO system, consisting of a VP-4450HD processor and an LL 4450 light source (FUJIFILM Co., Tokyo, Japan) (Video S1). Endoscopic video clips of the entire gastric mucosa were recorded for about a min using WLI, BLI-bright, and LCI after washing and removing gastric mucus (Figure 1a,b). At this time, we paid particular attention to keeping the shooting speed as constant as possible. Next, a close-up movie of the EGCs was recorded for several seconds using WLI, BLI-bright, and LCI. At this time, we recorded a movie of the entire lesion in the front view from a short or middle distance (Figure 1c,d,f). Then, low magnifying movies of EGC with the demarcation line at the oral or anal side of the lesion were recorded for several seconds using WLI and each IEE (Figure 1e).

### 2.5. The Evaluation of Endoscopic Findings

The evaluation of endoscopic findings was performed by 3 endoscopists who had performed more than 100 upper gastrointestinal endoscopies using the LASEREO endoscopic system. They had not received any information prior to the evaluation of the movies in this study. All of the movies were evaluated in random order.

The visibility of endoscopic findings, including the atrophic border, intestinal metaplasia, map-like redness on the remaining fundic gland, and the lesions, was evaluated using non-magnifying movies of approximately one min, and the visibility of the demarcation line (DL), microsurface (MS), and microvasculature (MV) of the lesions was evaluated using magnifying movies.

All endoscopic findings were evaluated for visibility using the following scores: 4, excellent visibility (easy detectable or recognizable); 3, good visibility (detectable or recognizable with careful observation); 2, fair visibility (detectable or recognizable with repeated careful examination; and 1, poor visibility (not detectable or not recognizable with repeated careful examination) [20]. Regarding the detection of the lesions, the evaluator was asked to watch the video and describe location of the lesion. If the lesion was missed or recognized at another site, the visibility score was rated as 1.

To obtain objective data, we compared the visibility of each IEE with that of WLI. The 31 cases evaluated by the 3 evaluators (93 cases) were divided into 3 categories concerning whether the visibility was improved (increase in visibility score), unchanged (the same score), or worsened (decrease in score) compared with WLI.

### 2.6. Definition of Endoscopic Findings

In this study, the endoscopic findings characteristic of gastritis using WLI were defined as follows: atrophic border, a discolored and thinning mucosa with a visible vascular pattern classified as mild or severe according to the Kimura–Takemoto classification [21]; intestinal metaplasia, multiple ashen nodular or cobblestone-like lesions observed typically on the atrophic mucosa; map-like redness, an erythematous lesion with a shallow depression and boundaries distinct from the background mucosa, in a variety of sizes and color tones according to previous reports [18]. The histological examination was not performed in this study to evaluate gastritis finding, as the endoscopists were the experts.

### 2.7. Diagnostic Criteria of Successful HP Eradication

The absence of HP infection with a personal history of HP eradication therapy was confirmed by at least one of the following reliable clinical tests: 13 C-urea breath test (UBit; Otsuka, Tokyo, Japan), stool antigen test, and a histopathological evaluation.

### 2.8. Calculation of the Sample Size

In a previous study, the mean visibility scores of BLI-bright, LCI, and WLI for 30 EGCs at distant observation were 3.50, 3.33, and 3.14, respectively [22]. Assuming the mean visibility score of the modality with the highest score to be 3.5, 3.3, and 3.1, respectively, and the standard deviation (SD) to be 1.0, a minimal sample size of 69 lesions was estimated to be necessary to reject the null hypothesis with a probability (power) of 0.8 and a probability of error (α) of 0.05. Therefore, a minimal sample size of 23 lesions with 3 endoscopists evaluating the visibility scores among each modality was needed.

### 2.9. Statistical Analyses

Quantitative data are expressed as the mean and SD. The mean visibility scores (MVSs) were calculated for the comparison of all pairs, and subjected to the Steel–Dwass test as a non-parametric multiple comparison. The relative visibility of each IEE to WLI was compared using Fisher’s exact test. With respect to the analysis of inter-observer variability, the Gwet’s AC1 values for the three endoscopists were calculated in WLI and each IEE [23]. *p* < 0.05 was considered statistically significant. All statistical analyses were performed using the JMP Pro version 15.2 (SAS International Inc., Cary, NC, USA) and R statistical software 3.6.2 (R core team, Vienna, Austria).

## 3. Results

### 3.1. Clinicopathological Features

**Figure 2:**
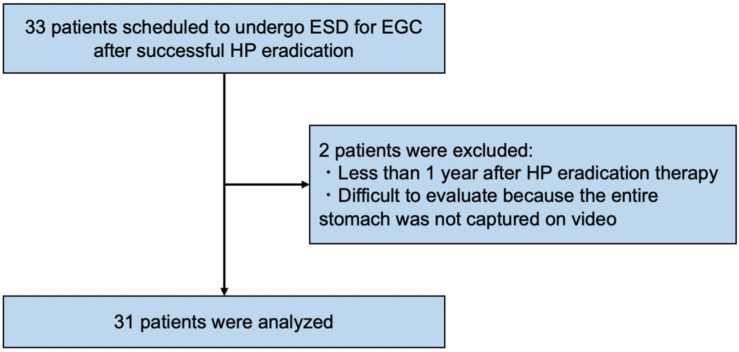
Flowchart of the study patients. ESD, endoscopic submucosal dissection; EGC, early gastric cancer; HP, *Helicobacter pylori*.

A total of 33 patients scheduled to undergo ESD for EGC after successful HP eradication in the four institutions were enrolled in this study. After enrollment in the study and video recording, two ineligible patients were excluded, and finally 31 patients (17 males, 14 females; mean age 72.1 years old) were evaluated in this study (Figure 2).

The clinicopathological features of all patients are shown in Table 1. Atrophic border was assessed according to the Kimura–Takemoto classification system; 3.2% had mild atrophy (C-1 and C-2), 48.4% had moderate atrophy (C-3 and O-1), and 48.4% had severe atrophy (O-2 and O-3). Of these 31 cases, intestinal metaplasia was present in 96.8%, and map-like redness in 48.4%. The pathological findings of lesions resected by ESD included 27 differentiated and four undifferentiated adenocarcinomas. The pathology of mixed differentiated and undifferentiated adenocarcinoma was defined as the predominant histological type.

### 3.2. The Visibility of EGC after Successful HP Eradication

**Figure 3:**
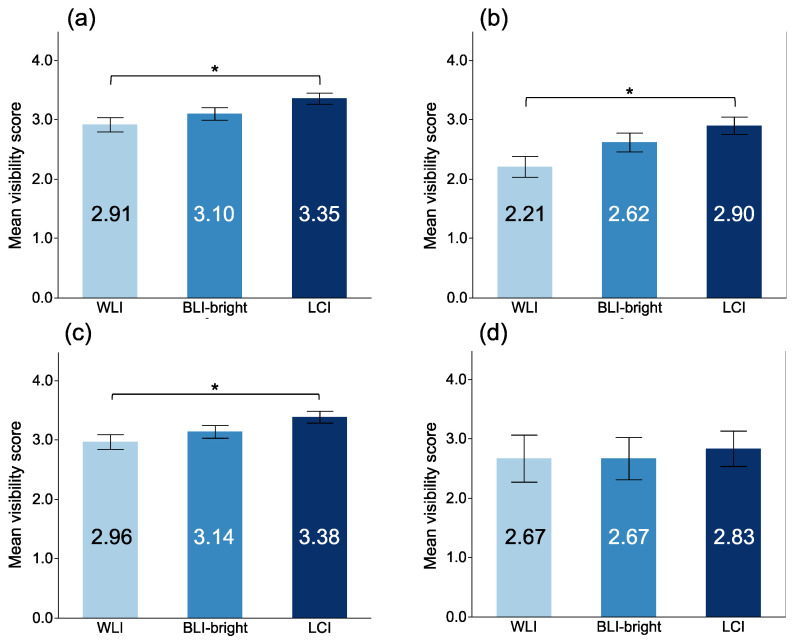
Mean visibility score of EGC among each modality in non-magnifying observation. (**a**) all EGCs; (**b**) EGC surrounded by map-like redness; (**c**) differentiated adenocarcinoma; (**d**) undifferentiated adenocarcinoma. *: *p* < 0.05.

The MVSs for EGC with each modality are shown in Figure 3. The MVSs with LCI for a non-magnifying view were significantly higher than those of WLI (3.35 ± 0.92, 2.91 ± 1.16, respectively, LCI vs. WLI: *p* = 0.022). However, there was no significant difference from BLI-bright (3.10 ± 1.02, *p* = 0.158). In 13 of the 31 lesions in this study, EGC was surrounded by map-like redness. Of these 13 lesions, eight had a reddish color, and the other five were discolored or had a normal mucosal color. LCI showed map-like redness with a lavender color, while EGC appeared orange. The MVSs with non-magnifying LCI in the 13 EGCs surrounded by map-like redness were significantly higher than those with WLI (2.90 ± 0.91, 2.21 ± 1.10, respectively, LCI vs. WLI: *p* = 0.012). There were significant differences between the MVSs with LCI and WLI in differentiated GC (3.38 ± 0.89 vs. 2.96 ± 1.12, respectively, *p* = 0.040). There were not significant differences between the MVSs with LCI and WLI in undifferentiated GC (2.83 ± 1.03 vs. 2.67 ± 1.37, respectively, *p* = 0.976).

**Figure 4:**
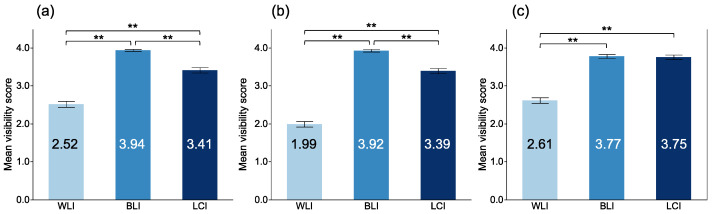
Mean visibility scores of EGC among modalities at magnifying observation. (**a**) microsurface at magnifying observation; (**b**) microvasculature at magnifying observation; (**c**) demarcation line at magnifying observation. **: *p* < 0.01.

The MVSs for a magnifying view with each modality are shown in Figure 4. The MVSs with BLI were the highest for DL, MV pattern (MVP), and MS pattern (MSP). There was no significant difference between BLI and LCI in the DL visibility (3.77 ± 0.49 vs. 3.75 ± 0.58, *p* = 0.974). However, the MVSs with BLI were significantly higher than those with LCI for MSP (3.94 ± 0.25 vs. 3.41 ± 0.68, *p* < 0.001) and MVP (3.92 ± 0.34 vs. 3.39 ± 0.64, *p* < 0.001).

### 3.3. The Visibility of Gastric Mucosal Findings after Successful HP Eradication

**Figure 5:**
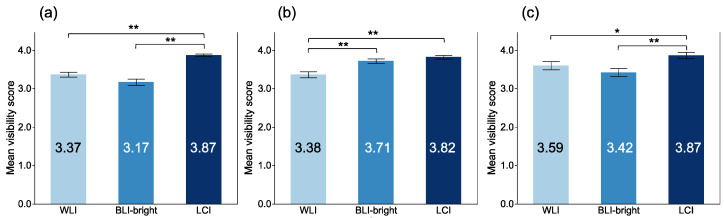
Mean visibility score of gastric mucosal findings among each modality. (**a**) atrophic border; (**b**) intestinal metaplasia; (**c**) map-like redness. *: *p* < 0.05, **: *p* < 0.01.

The mean visibility scores (MVSs) of atrophic border, intestinal metaplasia, and map-like redness among each modality are shown in Figure 5. The MVSs with LCI for atrophic border, intestinal metaplasia, and map-like redness were the highest among each modality, respectively. The MVSs with LCI for atrophic border, intestinal metaplasia, and map-like redness were 3.87 ± 0.34, 3.82 ± 0.49, and 3.87 ± 0.50, respectively. There were significant differences in atrophic border and intestinal metaplasia between LCI and WLI (*p* < 0.001, and *p* < 0.001, respectively).

### 3.4. Relative Evaluation of the Visibility

We compared the visibility of each IEE with that of WLI (Table 2). Regarding the visibility of gastric mucosa findings and EGC observed with a non-magnifying endoscope, LCI had a higher rate of improvement from WLI in the visibility of atrophic border, intestinal metaplasia, map-like redness, and EGC compared to BLI-bright (*p* < 0.001, *p* < 0.001, *p* < 0.001, and *p* < 0.001, respectively). In contrast, regarding the visibility of findings observed with a magnifying endoscope, BLI had a higher rate of improvement from WLI in the visibility of MSP, MVP, and DL compared to LCI ((*p* < 0.001, *p* < 0.001, and *p* < 0.001, respectively). Overall, there were very few cases in which visibility with LCI was worse than that with WLI.

### 3.5. Inter-Observer Variability

Regarding the analysis of inter-observer variability (Table 3), the mean of the Gwet’s AC1 values of gastric mucosa findings, and EGC observed with a non-magnifying endoscope, the observation with LCI showed good agreement between the observers (for atrophic border was 0.97, for intestinal metaplasia was 0.94, for map-like redness was 0.93, and for recognition of EGC was 0.85). Regarding the mean of the Gwet’s AC1 values for findings observed with a magnifying endoscope, the observation with BLI showed good agreement between the observers (for MSP was 0.99, for MVP was 0.97, and for DL was 0.93).

## 4. Discussion

This is the first study to evaluate the visibility of EGC and the Kyoto classification of gastritis after HP eradication using video clips with WLI, BLI/BLI-bright, and LCI. A comparative study was previously reported concerning the evaluation of EGC using still WLI, BLI, or LCI images [11,12]. However, there was a limitation of selection bias when using the best still images. Therefore, we evaluated the visibility using video clips with each modality near the clinical setting to avoid the selection bias associated with still images. In this study, the visibility score was evaluated as a subjective indicator because previous study demonstrated that this score was related to an objective indicator such as color difference value [24].

In our study, LCI showed the highest visibility of the Kyoto classification of gastritis, significantly for atrophic border, and intestinal metaplasia, but not significantly for map-like redness. Furthermore, LCI had the highest rate of improvement compared with WLI in the visibility of atrophic border, intestinal metaplasia, and map-like redness among IEE. These findings are attributed to LCI being image-processed with enough brightness to emphasize even slight color differences in the surface mucosa from a distant view [14]. Recently, the emergence of map-like redness was shown to be positive risk factors for EGC after successful HP eradication, and map-like redness was identified more frequently using LCI than WLI [18]. Although the histologic evaluation of each endoscopic finding was not performed in this study, at least in terms of map-like redness detection, LCI is expected to be the most suitable modality after HP eradication. In other words, LCI is considered the most suitable modality for identifying risks of EGC after HP eradication.

Excellent visibility of EGC after HP eradication was able to be secured using LCI instead of WLI. Furthermore, LCI had a higher rate of improved visibility of EGC compared with WLI than BLI-bright. We previously reported that BLI-bright improved the detection rate of EGC compared with WLI [9]. BLI-bright has limited colors to use to emphasize the structure of the mucosal surface, which may result in reduced brightness and visibility. In contrast, LCI, which has the same laser light settings as BLI-bright, has less restrictions on color and brightness than BLI-bright, thus maintaining a level of brightness that is as close as possible to that of WLI, making it easier to visualize EGCs even from a distant view. Therefore, LCI is expected to be most the suitable modality for detecting EGC after HP eradication, although the visibility was not markedly different between BLI-bright and LCI in our study.

EGC after HP eradication often shows slightly depressed and reddish lesions, known as a gastritis-like appearance [25]. It is particularly difficult to recognize EGC surrounded by map-like redness using WLI. However, LCI shows EGCs surrounded by map-like redness with greater detail than WLI, emphasizing the slight color difference between EGC and background map-like redness [26]. In our study, LCI showed EGC surrounded by map-like redness with a large color difference and better visibility than WLI. However, there were no significant differences in the visibility of undifferentiated EGC among modalities due to the small sample size in our study. Therefore, accumulating more cases will be necessary to prove the superiority of visibility with LCI for undifferentiated EGCs.

Regarding magnifying observation, the visibility score of BLI was significantly higher than that of other modalities. Furthermore, BLI had a higher rate of improvement from WLI in the visibility of MSP, MVP, and DL to LCI. This means that magnifying BLI is the most suitable modality for evaluating the MS and MV when determining the characteristics of EGCs. Given these results, the best observation method appears to be LCI for the entire stomach and then BLI for magnifying observation when EGCs are detected.

Several limitations associated with the present study warrant mention. First, this was a multi-center prospective study but had a small sample size. Second, there is a possibility of observer bias because observers could not be blinded to the type of IEE in the videos. Third, a cancerous lesion was included in all video clips. Fourth, there were fewer undifferentiated adenocarcinomas than differentiated adenocarcinomas. Fifth, there was a difference in the prevalence of each gastritis finding, and the sample size may not be sufficient for evaluate these findings.

In conclusion, LCI had the highest visibility among findings of the Kyoto classification of gastritis and EGC after HP eradication, and BLI had the highest visibility of MVP, MSP, and DL in magnifying observation. These results suggest that LCI observation in the entire stomach and further magnifying BLI are the best methods for detecting and diagnosing EGCs after HP eradication, respectively.

## Figures and Tables

**Table 1:** Clinicopathological features.

	*n* = 31
Age, years, median (range)	72.1 (61–85)
Sex, *n* (%)	
Male	17 (54.8)
Female	14 (45.2)
Time since H pylori eradication, y	5.58
Atrophic border, *n* (%)	data
C-1·C-2	1 (3.2)
C-3·O-1	1 (3.2)
O-2·O-3	29 (93.5)
Intestinal metaplasia, *n* (%)	
−	16 (51.6)
+	15 (48.4)
Location, *n* (%)	
U	1 (3.2)
M	20 (64.5)
L	10 (32.3)
Macroscopic type, *n* (%)	
0-IIa	14 (45.2)
0-IIb	2 (6.5)
0-IIc	15 (48.4)
Mean tumor size, mm (range)	21.2 (10–50)
Color, *n* (%)	
Normal	2 (6.5)
Reddish	22 (71.0)
Discolored	7 (22.6)
Histological type, *n* (%)	
Differentiated	27 (87.1)
Undifferentiated	4 (12.9)

**Table 2:** Relative visibility of each IEE to WLI, IEE, image-enhanced endoscopy; WLI, white light imaging; BLI, blue laser imaging; LCI, linked color imaging; EGC, early gastric cancer.

		Relative Visibility, *n* (%)		*p*-Value
		BLI-Bright/BLI	LCI	
Atrophic border	Improved	9 (9.7)	42 (45.2)	<0.001
	Unchanged	58 (62.4)	51 (53.8)	
	Worsened	26 (28.0)	0	
Intestinal metaplasia	Improved	31 (33.3)	35 (37.6)	<0.001
	Unchanged	58 (62.4)	57 (61.3)	
	Worsened	4 (4.3)	1 (1.1)	
Map-like redness	Improved	11 (11.8)	23 (24.7)	<0.001
	Unchanged	64 (68.8)	70 (75.3)	
	Worsened	18 (19.4)	0	
Recognition of EGC	Improved	21 (22.6)	34 (36.6)	<0.001
	Unchanged	62 (66.7)	58 (62.4)	
	Worsened	10 (10.8)	1 (1.1)	
Microsurface	Improved	86 (92.5)	66 (71.0)	<0.001
	Unchanged	7 (7.5)	25 (26.9)	
	Worsened	0	2 (2.2)	
Microvascular	Improved	90 (98.9)	76 (81.7)	<0.001
	Unchanged	3 (3.2)	16 (17.2)	
	Worsened	0	1 (1.1)	
Demarcation line	Improved	85 (91.4)	85 (91.4)	<0.001
	Unchanged	8 (8.6)	8 (8.6)	
	Worsened	0	0	

**Table 3:** Inter-observer variability (Gwet’s AC1). WLI, white light imaging; BLI, blue laser imaging; LCI, linked color imaging; EGC, early gastric cancer.

Inter-Observer Variability, Mean (Range)	
Atrophic border	
WLI	0.78 (0.67–0.86)
BLI-bright	0.62 (0.50–0.79)
LCI	0.97 (0.96–0.98)
Intestinal metaplasia	
WLI	0.61 (0.35–0.91)
BLI-bright	0.88 (0.87–0.90)
LCI	0.94 (0.92–0.96)
Map-like redness	
WLI	0.73 (0.60–0.93)
BLI-bright	0.75 (0.67–0.84)
LCI	0.93 (0.88–0.97)
Recognition of EGC	
WLI	0.74 (0.72–0.76)
BLI-bright	0.69 (0.62–0.74)
LCI	0.85 (0.84–0.86)
Microsurface	
WLI	0.78 (0.75–0.82)
BLI	0.99 (0.98–0.99)
LCI	0.84 (0.78–0.93)
Microvascular	
WLI	0.70 (0.53–0.84)
BLI	0.97 (0.95–1.00)
LCI	0.79 (0.72–0.89)
Demarcation line	
WLI	0.78 (0.68–0.84)
BLI	0.93 (0.89–0.96)
LCI	0.88 (0.84–0.97)

## Data Availability

Data will be available from the corresponding author upon reasonable request.

## Supplementary Materials

The following are available online at https://www.mdpi.com/article/10.3390/jcm10163649/s1, Video S1: Video clip of the endoscope actually used for the visibility evaluation. Endoscopic video clips of the entire gastric mucosa were recorded for about a min using WLI, BLI-bright, and LCI. Next, a close-up movie of the EGCs was recorded for several seconds using WLI, BLI, and LCI. At this time, we recorded a movie of the entire lesion in the front view from a short or middle distance. Then, low magnifying movies of EGC with the demarcation line at the oral or anal side of the lesion were recorded for several seconds using WLI and each IEE.
